# Multi-Approach Analysis Reveals Pathways of Cold Tolerance Divergence in *Camellia japonica*

**DOI:** 10.3389/fpls.2022.811791

**Published:** 2022-02-25

**Authors:** MengLong Fan, Ying Zhang, XinLei Li, Si Wu, MeiYing Yang, Hengfu Yin, Weixin Liu, Zhengqi Fan, Jiyuan Li

**Affiliations:** Research Institute of Subtropical Forestry, Chinese Academy of Forestry, Hangzhou, China

**Keywords:** cold, transcriptome, proteome, plant hormone, co-expression, *Camellia japonica*

## Abstract

Understanding the molecular mechanism of the cold response is critical to improve horticultural plant cold tolerance. Here, we documented the physiological, transcriptome, proteome, and hormonal dynamics to cold stress in temperate genotype (Tg) and subtropical genotype (Sg) populations of *Camellia japonica*. Tg *C. japonica* suffered minimal osmotic and oxidative damage compared to Sg *C. japonica* under the same cold treatment. Transcriptional and translational differences increased under the cold treatment, indicating that Tg *C. japonica* was affected by the environment and displayed both conserved and divergent mechanisms. About 60% of the genes responding to cold had similar dynamics in the two populations, but 1,896 transcripts and 455 proteins differentially accumulated in response to the cold between Tg and Sg *C. japonica*. Co-expression analysis showed that the ribosomal protein and genes related to photosynthesis were upregulated in Tg *C. japonica*, and tryptophan, phenylpropanoid, and flavonoid metabolism were regulated differently between the two populations under cold stress. The divergence of these genes reflected a difference in cold responsiveness. In addition, the decrease in the abscisic acid (ABA)/gibberellic acid (GA) ratio regulated by biosynthetic signal transduction pathway enhanced cold resistance in Tg *C. japonica*, suggesting that hormones may regulate the difference in cold responsiveness. These results provide a new understanding of the molecular mechanism of cold stress and will improve cold tolerance in horticultural plants.

## Introduction

Cold stress is a major abiotic factor that limits the distribution and growth of plants (Theocharis et al., 2012). The high complexity of the plant’s response to cold makes the task of identifying causal loci, alleles, and genes challenging. Cold stress has been divided into chilling (0–15°C) and freezing (< 0°C) stressors (Thomashow, 1999). Freezing promotes the movement of water from the cell into the extracellular space. Plants withstand cold stress by producing supercooling compounds or controlling the rate of ice growth (Ninagawa et al., 2016). The freezing process usually triggers a more intense and comprehensive physiological and molecular response, and a large number of direct or indirect pathways affect the response of plants to cold (Maynard et al., 2018). A series of regulators have been isolated and identified, including signaling molecules (Ca^2+^), a cold-resistant protein, and transcription factors (Wang et al., 2019). Cold stress reduces photosynthetic ability, and promotes the activation of ascorbate peroxidase in rapeseed (Mehmood et al., 2021). Phosphoenolpyruvate carboxylase (PEPC) and malate dehydrogenase are involved in the photosynthetic pathway and are regulators of cold tolerance in *C. japonica* (Fan et al., 2021a). Furthermore, the ribosome is a regulator of the cold response and correlates with the biosynthesis of many cold-resistant proteins (Dong et al., 2002; Yu et al., 2020). REIL proteins affect cold-induced plants by ribosome remodeling, and are required for the accumulation of the 60s ribosome (Beine-Golovchuk et al., 2018). Plant hormones play important roles in the regulation of stress (Fadon et al., 2020). Avocados undergo a quick response to cold stress leading to changes in abscisic acid (ABA) and jasmonates (Vincent et al., 2020). Moreover, brassinosteroids (BRs) enhance freezing tolerance, in part, by activating the *CBF-COR* pathway (Li H. et al., 2017). Plants respond to cold stress by regulating the processes of transcription and translation. Transcriptional and post-translational modification are widely involved in stress. Transcriptome and proteomic data provide insight into these changes that occur under cold stress (Dai et al., 2021).

Different populations of the same species establish different gene regulated networks, protein recombinations, physiological functions, and heritable changes to adapt to new needs under long-term selection pressures (Li et al., 2021). The process involves the allocation of limited resources within the plant (Olsen et al., 2016; Hahn et al., 2019). Comparative analysis of peanut varieties with different cold tolerances suggests that genes related to the soluble sugar and lignin biosynthetic pathways play an important role in differences in cold responsiveness (Wang X. et al., 2021). Thus, it is crucial to focus on the differences in cold resistance caused by natural domestication.

The genus *Camellia* contains about 250 species, which provide value for the pharmaceutical, food, art, and landscape industries (Huang et al., 2017). Most of these species are cold-sensitive and are distributed in the subtropics. *C. japonica* (Naidong) is a woody ornamental plant and the only camellia species with a temperate-zone genotype (Tg) in China (Fan et al., 2021a). *C. japonica* is a Tertiary relic species that experienced large changes in temperature during the Quaternary Ice Age. The Tg population in Qingdao, China (36°14′N, 120°46′E) adapted to the cold and was preserved, while most of the other populations are concentrated near Zhoushan, China (subtropical zone), as well as in Japan and Korea. *C. japonica* (Naidong) is an ancient species with representative genetic characteristics (Wang et al., 2007; Li et al., 2013). However, camellias lack molecular resources, and research focusing on the *C. japonica* response to cold is rare. Thus, it is important to explore the cold resistance mechanism in camellia.

Based on the heterogeneity of the growing environment and previous studies, we postulated that Tg *C. japonica* would have a higher resistance to cold than Sg *C. japonica*, and would provide cold tolerance capacity that is absent in the camellia gene pool. In the present study, we compared the phenotypes and physiology of Tg and Sg *C. japonica* under cold stress. As results, Tg *C. japonica* physiologically behaved like a cold-tolerant plant. Combining the transcriptome, proteomics, and hormonal data revealed the molecular divergence between Tg and Sg *C. japonica*. Interestingly, the cold treatment was transcriptionally more differentiated from the control. We assumed that the cold resistance genes and pathways were strongly differentially regulated between Tg and Sg and thus would maintain the growth of *C. japonica* under cold conditions. Our results reveal a putative model of differential cold tolerance. This model includes genes acting in photosynthesis, secondary metabolism, and hormone-related pathways, which function to modify cold resistance in *C. japonica*.

## Results

### Physiological Differences Between Tg and Sg *Camellia japonica* in Response to Cold Stress

**FIGURE 1:**
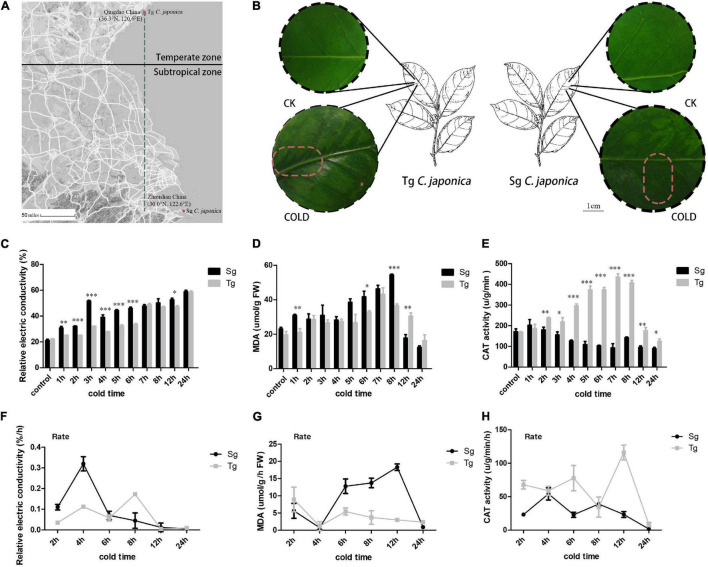
Divergence of cold tolerance between temperate (Tg) and subtropical (Sg) *C. japonica*. **(A)** Distributions of the two *C. japonica* populations. The green line represents the latitudinal difference between the Sg and Tg populations. The raw map was downloaded from Google Maps (https://www.google.com/maps) **(B)** phenotypic changes in leaves under the 24 h cold treatment. The dark area represents the accumulation of cold damage. **(C–E)** Indicate the content of relative electric conductivity, MDA, and CAT activity, respectively. **(F–H)** Indicate changes in the rate of relative electric conductivity, MDA, and CAT activity, respectively. Data are mean ± *SD* of three independent experiments, * indicates a significant difference at *P* < 0.05 between Tg and Sg *C. japonica* at a particular time by Student’s *t*-test. ^**^p < 0.01, ^***^p < 0.001.

Tg and Sg *C. japonica* were selected based on latitude (Figure 1A), inferring differences in cold sensitivity. The local environmental temperature of Tg plants is lower than that of Sg plants, indicating that Tg might harbor cold-tolerant traits. Here, the two groups of plants were exposed to the same cold treatment of -5°C for 0, 1, 2, 3, 4, 5, 6, 7, 8, 12, and 24 h. Sg *C. japonica* suffered more damage than Tg *C. japonica* (Figure 1B and Supplementary Figure 1), based on malondialdehyde (MDA) and relative electrical conductivity measurements. Tg *C. japonica* had less osmotic and oxidative damage than Sg *C. japonica* (Figures 1C,D and Supplementary Table 1). Tg *C. japonica* had higher H_2_O_2_ catalase (CAT) activity than Sg *C. japonica* (Figure 1E). In contrast, a significant difference in the rate of change in electrical conductivity was detected between the two populations at 4 and 8 h (Figure 1F and Supplementary Table 1). The 4–24 h time interval revealed significant differences in MDA and CAT levels (Figures 1G,H). Thus, the transcriptome was sequenced using the 0 (CK), 4 (T1), 8 (T2), and 24 h (T3) cold treatments.

### Establishing the RNA and Protein Libraries of the Two *Camellia japonica* Natural Populations Under Cold Stress

More than 168.23 Gb of clean data were collected from 24 samples with Q30 > 85.1% (Supplementary Table 2). The clean reads ranged from 20,404,743 to 31,731,765. Then, 49,394 unigenes were obtained using Trinity software. Furthermore, 5,855 proteins were quantified in the proteome. The Pearson’s correlation coefficients for the biological replicates of the different samples varied from 0.82 to 0.87 (Supplementary Table 2). These results indicate that the throughput and sequencing quality was sufficient to warrant further analysis.

### Basic and Cold Treatment Transcriptome and Proteome Divergence Between Tg and Sg *Camellia japonica*

**FIGURE 2:**
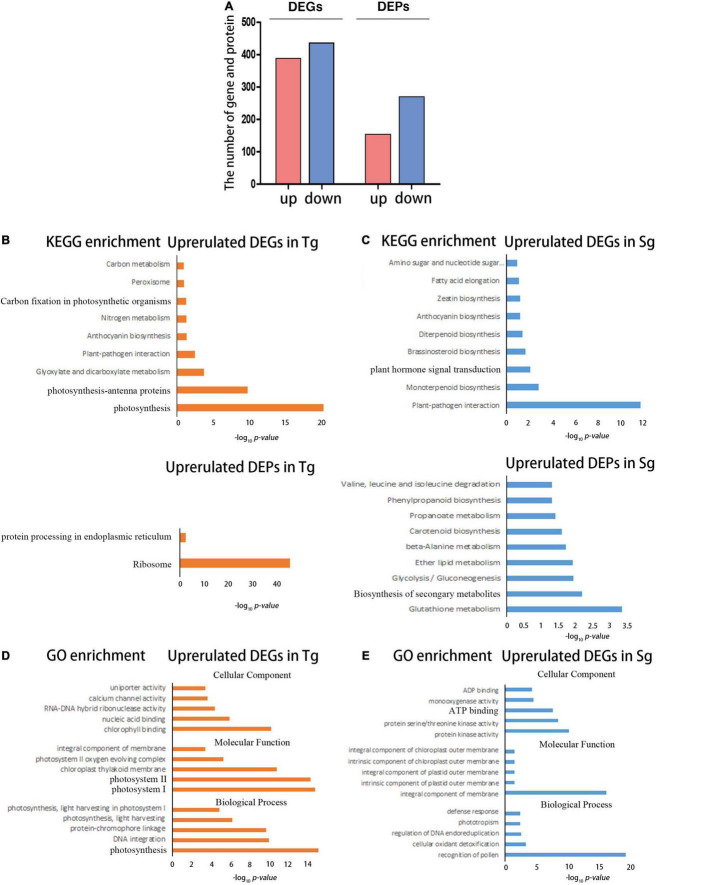
The transcriptome and proteome reflecting the differences between Tg and Sg *C. japonica* under normal conditions. **(A)** Number of DEGs and DEPs under the control temperature conditions. **(B)** The bar chart shows the significantly enriched KEGG pathways for the highly expressed DEGs and DEPs in Tg *C. japonica*, The abscissa represents the -log_10_ (*p*) value. **(C)** The bar chart shows the significantly enriched KEGG pathways for the highly expressed DEGs and DEPs in Sg *C. japonica*. The abscissa represents the -log_10_ (*p*) value. **(D)** The plot shows the significantly enriched GO terms for higher expression of DEGs and DEPs in Tg *C. japonica*, and the abscissa represents -log_10_ (*p*) value. **(E)** Plot shows the significantly enriched GO terms for the higher expression of DEGs and DEPs in Sg *C. japonica*. The abscissa represents -log_10_ (*p*) value.

We focused on the differences in transcriptome and proteome expression between the normal temperature treatments (control and unstressed). This approach further highlighted the significant differences in basic transcription. The Sg population was taken as the control. The screening condition was set to fold-change ≥ 2 and a false discovery rate (FDR) < 0.01 to identify differentially expressed genes (DEGs). In the Sg vs. Tg comparison, 816 genes were identified as DEGs; 384 genes were upregulated and 432 genes were downregulated. The screening conditions were set to fold change ≥ 1.5 and an FDR < 0.01 to identify differentially expressed proteins (DEPs). As results, 149 proteins were upregulated and 265 proteins were downregulated (Figure 2A).

The DEGs and DEPs were functionally enriched. The photosynthesis, ribosome-related genes, and proteins exhibited higher expression levels in Tg than in Sg *C japonica* (Figure 2B and Supplementary Table 3). Plant hormone signal transduction, plant-pathogen interactions, and biosynthesis of secondary metabolite pathways, such as flavonoid and anthocyanin biosynthesis-related genes and proteins, were expressed at higher levels in Sg than Tg *C. japonica* (Figure 2C and Supplementary Table 3). In addition, Gene Ontology (GO) enrichment analysis was performed, and terms such as photosynthesis-related “photosystem I and II,” “integral component of the membrane,” and “nucleic acid binding” were enriched in Tg (Figure 2D and Supplementary Table 4). Similarly, the regulation of DNA endoreduplication, serine/threonine kinase activity, and terpene synthase activity were enriched in Sg *C. japonica* (Figure 2E and Supplementary Table 4).

**FIGURE 3:**
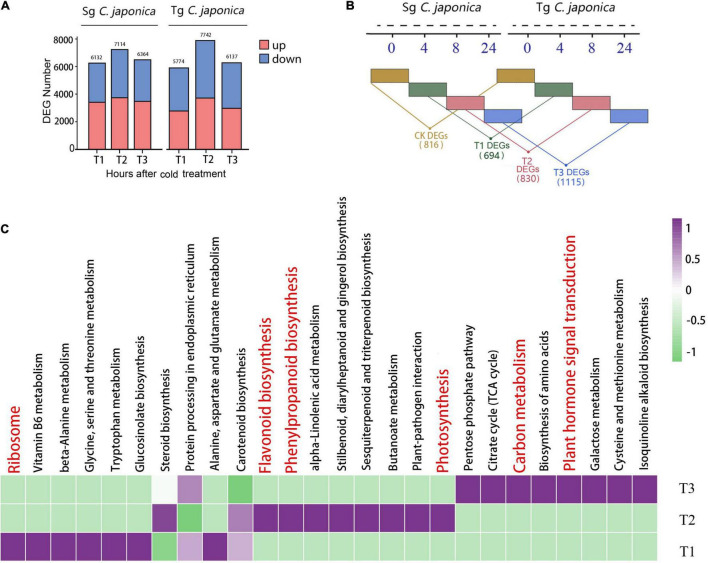
Divergence in the response to cold between Tg and Sg *C. japonica*. **(A)** The number of DEGs and DEPs after the T1, T2, and T3 cold treatments. **(B)** Representation of DEGs between two camellias under the same cold treatment. Numbers in parentheses show the number of DEGs within a category. **(C)** Top 26 enriched KEGG pathways of these three groups.

The transcriptomic data during the cold treatment showed that Tg and Sg *C. japonica* displayed similar transcriptional dynamics. Interestingly, in the T1 and T3 treatment groups, the number of DEGs in Tg *C. japonica* was lower than in the Sg. In the T2 treatment group, both Sg and Tg displayed the largest number of DEGs, as 7,114 and 7,742 genes were identified as DEGs, respectively (Figure 3A). Furthermore, the iTRAQ proteome of the T2 treatment group was identified. Relative to the control, 55 upregulated proteins and 27 downregulated proteins were detected in Tg *C. japonica* treated at −5°C for 8 h. Seventy-three upregulated proteins and 49 downregulated proteins were detected in Sg *C. japonica* during the same treatment. KEGG functional enrichment analysis showed that these DEGs and DEPs were enriched in the ribosome, photosynthesis-related, protein processing in the endoplasmic reticulum, plant hormone signal transduction, glycerophospholipid metabolism, and secondary metabolic pathways (Supplementary Table 5). In addition, these data suggest a broad restructuring of the proteome as a common response to cold stress.

We focused on a pairwise comparative analysis of the two populations under the same cold treatment to explore the differences in cold responsiveness. The pairwise comparative results suggested that 816, 694, 830, and 1,115 DEGs were identified at the CK, T1, T2, and T3 treatments, respectively (Figure 3B), and displayed larger differences between the populations than the control. These results indicate that marked interspecific variation and basal transcription were more conserved among the tested accessions than cold-related transcription. A total of 166 genes were shared by the two populations during cold stress, and 431 genes were specific to the T2 group (Supplementary Figure 2). The KEGG pathway enrichment analysis revealed numerous pathways related to carbon metabolism, biosynthesis of secondary metabolites (flavonoids and phenylpropanoids), and protein synthesis processing that were common and upregulated in Tg *C. japonica*, including protein processing in the endoplasmic reticulum, ribosome, and ribosome biogenesis, and photosynthesis and phenylpropanoid metabolism (Figure 3C and Supplementary Table 6).

**FIGURE 4:**
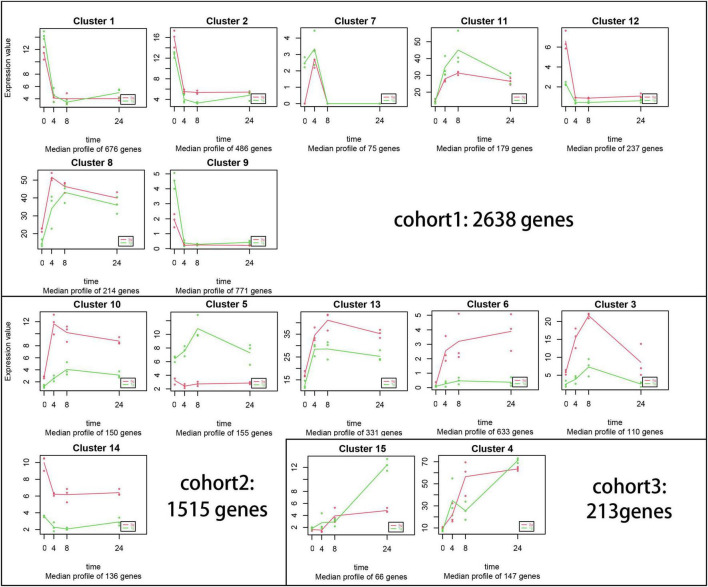
Gene co-expression and cluster analyses using MaSigPro software. Each plot represents similarly expressed genes during the cold treatment (0, 4, 8, and 24 h). *R*^2^ > 0.6. These plots are grouped into cohorts 1, 2, and 3 according to the expression level.

An expression pattern analysis was performed across all treatments and populations using MaSigPro software (Figure 4). A total of 4,366 genes were divided into 15 clusters (*R*^2^ ≥ 0.6, adjusted *p* < 0.05, Figure 4). Five of the 15 clusters included population-associated differences in the intensity of expression for 1,374 (31%) genes, independent of cold stress time. Cohort 1 included 2,638 genes with similar expression levels under cold stress, while cohort 2 included 1,515 (34%) genes with distinctly higher or lower expression in only a single population. Cluster 5 was the only cluster with higher Tg than Sg expression levels. The expression patterns of 213 (4%) genes were different or opposite (cohort 3), indicating that the expression differences could be transmitted across the domestication gradient.

### Identification of Gene Co-expression Modules Related to the Divergence in Cold Responsiveness

**FIGURE 5:**
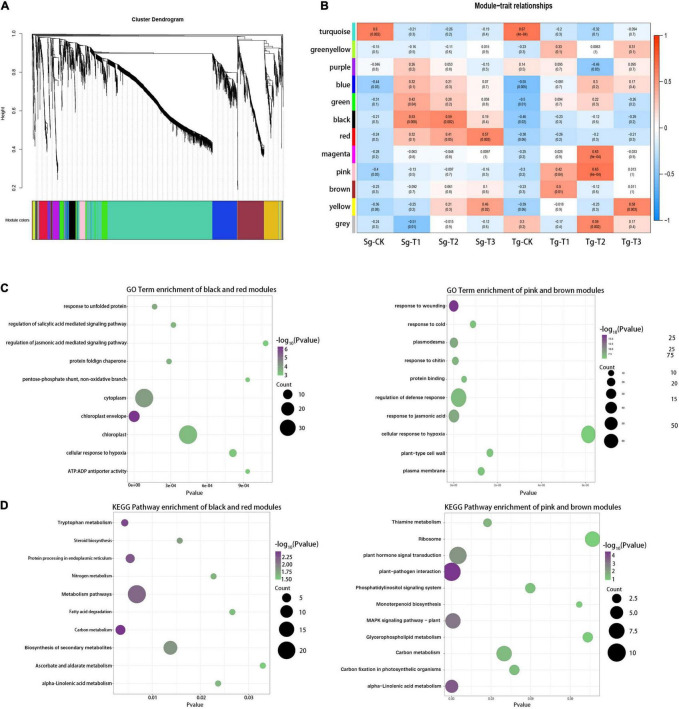
Weighted gene network analysis of the response of the genes to cold. **(A)** Twelve modules of co-expressed genes are shown on the hierarchical cluster tree. Each branch represents a DEG. **(B)** Correlations of the gene expression modules, the two populations, and the 0 (CK), 4, 8, and 24 h treatments. Red and blue indicate the positive and negative correlations, respectively. The correlation coefficient and the *p*-value are shown within each cell. **(C)** Top 10 enriched GO terms of the Sg and Tg *C. japonica* modules within the highest correlation; bubble color indicates the -log10(*p*) value of the enriched GO term. **(D)** Top 10 enriched KEGG pathways of the Sg and Tg *C. japonica* modules with the highest correlation; bubble color indicates the -log10 (*p*) value of the enriched KEGG pathways. The pathways of concern are marked in red.

To obtain the hub components associated with the differences in cold responsiveness, 3,119 genes with the largest contribution to 25% of the variance were screened from 12,475 DEGs for the weighted gene co-expression network analysis (WGCNA). The Power 8 network topology was selected to construct a hierarchical cluster tree. All genes were clustered into 12 modules (labeled with different colors, Figure 5A). All of the modules were highly correlated with the respective module. We associated each co-expression module with a cold treatment time in the two populations using Pearson’s correlation coefficient analysis (Figure 5B). The turquoise module was correlated with the control groups of both populations, but the black and red modules were correlated with the Sg-T2 and Sg-T3 groups, respectively (*r* = 0.59, *p* = 2e −3 and *r* = 0.65, *p* = 6e −4). The correlation between the pink module and the Tg-T2 group was the highest (*r* = 0.57, *p* = 3e −3), and the brown modules were correlated with the Tg-T1 group (*r* = 0.5, *p* = 3e −3). The transcriptional difference caused by the cold treatment was greater than that caused by the different populations. The difference among the populations under the cold treatment was greater than that under normal growth, indicating that *C. japonica* (Naidong) formed conservative and divergent evolution caused by environmental differences, and Tg *C. japonica* was more radical against cold stress.

GO and KEGG analyses were performed for the gene modules mirroring the divergence in cold responsiveness. The red and black modules contained the specific genetic information for the response to cold stress in Sg *C. japonica*, and the brown and pink modules represented that for Tg *C. japonica*. The first 30 enriched GO terms (*p* < 0.05) showed that terms related to protein processes were enriched in the black and red modules, e.g., “response to unfolded protein,” “protein-folding chaperone,” and “protein storage vacuole.” In addition, many terms were related to the chloroplast, e.g., “chloroplast envelope” and “chloroplast.” Interestingly, the degree of enrichment of terms related to the response to a stimulus was higher in the brown and pink modules than in the red and black modules, e.g., “response to cold,” “response to jasmonic acid,” and “regulation of defense response.” In addition, “plasmodesma” and “DNA-binding transcription factor activity” were also enriched (Figure 5C and Supplementary Table 7). The KEGG enrichment analysis (*p* < 0.05) suggested that the ribosome, plant hormone signal transduction, photosynthesis-related, glycerophospholipid metabolism, and plant-pathogen interaction pathways were significantly enriched in Tg *C. japonica*, while protein processing in the endoplasmic reticulum and biosynthesis of secondary metabolites were significantly enriched in Sg *C. japonica* (Figure 5D and Supplementary Table 8), suggesting that the Tg and Sg proteomes’ response to cold stress is broad. Overall, these findings are consistent with the results obtained, demonstrating that our results are reliable.

### Photosynthetic Pathway Response to the Divergence in the Cold Stress Response Between Tg and Sg *Camellia japonica*

Key candidate genes were screened from the transcriptome data based on standard gene names in the functional annotation of the pathways. Sixty-two and 64 DEGs were involved in the photosynthetic response to cold in Sg and Tg *C. japonica*, respectively (Supplementary Table 9). Thirty-two genes displayed different expression levels in the Sg vs. Tg comparison. Among these genes, six were specific to Tg, including *CjFD3* (c153759.graph_c0), *CjPSBR* (c129109.graph_c0), *CjFNR2* (c143865.graph_c0), *CjATPC1* (c146541.graph_c0), *CjPetA* (c148557.graph_c2), and *CjCF0* (c105617.graph_c0). Furthermore, 15 genes involved in carbon fixation during photosynthesis were identified as DEGs, e.g., glyceraldehyde-3-phosphate dehydrogenase (c117808.graph_c0) and ribulose bisphosphate carboxylase small chain (c122766.graph_c0) (Supplementary Table 10). To confirm the reliability of the transcriptome data, we selected five genes from these DEGs and validated them using quantitative real-time polymerase chain reaction (RT-qPCR). The expression trends in the RT-qPCR results were largely consistent with the transcriptome data (Supplementary Figure 3), suggesting that our data were reliable. These results indicate that these genes play an important role in photosynthesis regulated by local cold adaptation.

The differences in the transcriptomic and proteomic data were larger at the transcript level than at the protein level. Four photosynthesis-antenna proteins and five proteins involved in carbon fixation in photosynthetic organisms were identified as DEPs between Tg and Sg *C. japonica* under cold stress, e.g., glyceraldehyde-3-phosphate dehydrogenase B, transketolase, chloroplastic malate dehydrogenase, glyoxysomal, malate dehydrogenase, NADP-dependent malic enzyme-like, phosphoglycerate kinase, cytosolic, glyceraldehyde-3-phosphate dehydrogenase, photosystem I chlorophyll a/b-binding protein 3-1, and chlorophyll a-b binding protein CP29.1 (Supplementary Table 9). These results between Tg and Sg *C. japonica* at the translational level provide a stronger photosynthetic response to cold in Tg *C. japonica* than Sg *C. japonica.*

### Restructuring of the Proteome in Response to Cold

We focused on the divergence in ribosomes between Tg and Sg *C. japonica* during the cold treatment. Eighty-five genes displayed significantly different expression levels in response to cold stress between the two populations, including encoding large subunit ribosomal protein and small subunit ribosomal protein genes (Supplementary Table 11). Most of these genes were expressed at higher levels in Tg than Sg *C. japonica*; 56 DEPs involved in divergence in the ribosomal pathway between Tg and Sg *C. japonica* were screened in the proteome, e.g., 60S ribosomal protein L7a-1-like (c26982_f1p21_1112), 40S ribosomal protein S7 (c35197_f1p2_1028), 30S ribosomal protein S3 (chloroplast) (c16401_f1p4_4386), 50S ribosomal protein L27, chloroplastic (c74584.1_c1_orf5), and ribosomal protein S12 (chloroplast) (c75116.1_c0_orf7) (Supplementary Table 6). Consistent with the transcriptome results, only 30S ribosomal protein S5 and chloroplastic-like (c31055_f2p1_1207) were expressed at lower levels in Tg than Sg *C. japonica* (Supplementary Table 11). Taken together, the ribosome response to cold was stronger in Tg than Sg *C. japonica*, and protein processes played an important role.

### The *Camellia japonica* Hormone Network Under Cold Stress

**FIGURE 6:**
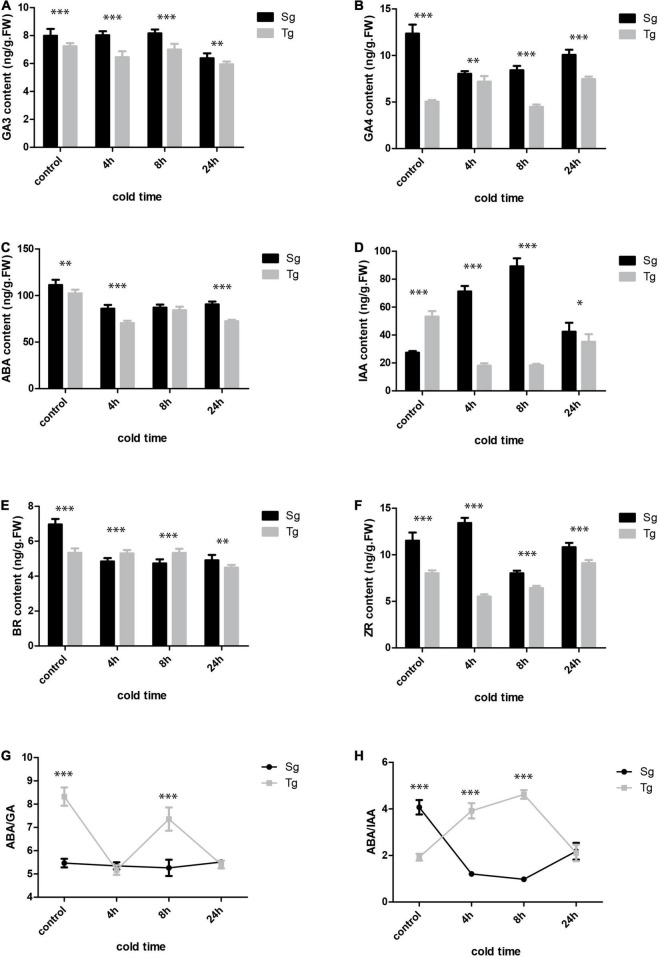
The hormone levels in the two *C. japonica* populations at 0, 4, 8, and 24 h of cold treatment time. **(A)** GA3. **(B)** GA4. **(C)** ABA. **(D)** IAA. **(E)** BR. **(F)** ZR. **(G)** ABA/GA ratio. **(H)** ABA/IAA ratio. Data are mean ± standard deviation (SD) (three biological replicates and three technical repeats), * symbol indicates significant difference between the two groups was determined by Student’s *t*-test (*P < 0.05, ^**^P < 0.01 or ^***^P < 0.001).

The hormone network plays a key role in the plant’s response to stress. We carried out a targeted hormone quantification analysis specifically targeting gibberellic acid (GA), ABA, auxin, BRs, and zeatin (ZR) (Figures 6A–F). The contents of these hormones were higher in Sg than in Tg *C. japonica*. GA3 was downregulated in the two *C. japonica* populations under cold stress, whereas GA4 was upregulated initially and then downregulated in Tg under cold stress, but displayed the opposite trend (downregulated first and then upregulated) in Sg *C. japonica* (Figures 6A,B). The indole acetic acid (IAA) levels were similar. Surprisingly, ABA was downregulated first and then upregulated in leaves in the 24-h -5°C treatment in both populations. Interestingly, the ABA/IAA ratios decreased in Sg *C. japonica* under cold stress, and ABA/GAs ratios decreased in Tg *C. japonica* under cold stress (Figures 6G,H), while the ABA/GA ratio did not change in Sg *C. japonica*, suggesting that Tg *C. japonica* has higher adaptability to the cold (Supplementary Table 12).

## Discussion

Cold is devastating stress based on its consequences during rapid climate change. Temperature represents a major factor determining the growth and production of camellia plants (Wisniewski et al., 2014; Fan et al., 2021a). We explored the divergence in the physiological, transcriptional, protein, and hormonal responses to cold stress between Tg and Sg *C. japonica* (Naidong) to identify the cold-responsive mechanisms under natural domestication. Comparing the Tg and Sg *C. japonica* populations allowed us to identify genes that could be immediate targets for breeding cold tolerance. Interspecific divergence may underlie the unique cold adaptations in camellia.

### Selective Environmental Pressure Causes the Differences in Cold Tolerance

Environmentally related differences in leaf morphology generally reflect the adaptive evolution of plants (Ordonez et al., 2009). The color and volume of leaves often mutate with a change caused by environmental stress (Givnish, 1978; Wright et al., 2005). No differences in leaf morphological traits were observed between the two populations of *C. japonica* under normal conditions. We cannot be sure whether this was affected by natural domestication because of the complexity of the climate conditions. Combining different types of data makes it possible to contrast the results and refine current hypotheses to provide a better understanding of the differences in cold tolerance.

As we speculated, the Tg plants suffered less osmotic and oxidative damage under the −5°C cold treatment than Sg plants. This finding is supported by the CAT activity results. The difference in latitude caused a large temperature difference, which promoted the difference in cold tolerance. The temperature is < 0°C for most of the winter in Qingdao, Shandong, and > 0°C for most of the winter in Zhoushan, Zhejiang (Supplementary Table 11). In our preliminary experiment, the same index was measured during the 4 and 0°C treatments, and no difference was observed between the two populations, indicating that plants evolve different cold-responsive mechanisms when populations grow under different cold stress intensities. Gene expression must change to modify physiological status and adapt to local environmental change; however, little is known regarding the molecular mechanism. Differences between populations of forsythia and *Coffea mauritiana* have been explored by transcriptome analyses (Garot et al., 2021; Li et al., 2021)Garot.

### Inferring Basic Transcriptional Divergence Caused by Different Plant Hormone Levels and Photosynthetic Abilities

Variations in the transcriptome mirror genetic variations among species (von Wettberg et al., 2018). High genetic diversity has been reported in *C. japonica* (Li et al., 2013). Our results revealed significant differences in the transcript levels between the Tg and Sg populations under an unstressed (normal) condition. Genes related to photosynthesis contributed significantly to the changes in the transcriptome, with higher expression levels in the Tg than the Sg population, including photosystem II (PS II), photosystem I (PS I), ATP synthase, and chlorophyll a/b-binding proteins. The proteomic results were similar. These differences could reflect differences in light utilization efficiency or growth ability between Tg and Sg *C. japonica*. A study on differences in cold responsiveness in forsythia confirmed that the high latitude population has higher carboxykinase ATP activity (Li et al., 2021). The expression level of genes related to hormone signaling was lower in Tg than Sg *C. japonica*. Our hormone profile confirmed this result. The BR, ABA, ZR, and GA levels were lower in Tg *C. japonica* than those in Sg. Interestingly, the IAA level in Tg *C. japonica* was higher, and some genes related to the biosynthesis of auxins were upregulated. ABA and GA play opposite roles in regulating growth (Shi et al., 2021). Overall, Tg *C. japonica* displayed stronger viability than Sg *C. japonica*, which may reflect differences in cold tolerance affected by basic transcription.

### Identification of the Core Conserved Part of the Stress Response to Cold

Despite differences in cold resistance and gene expression levels, both *C. japonica* populations displayed their largest transcriptional responses after 8 h of cold treatment. Combining functional enrichment of the transcriptome and proteome suggested that a range of conserved pathways responded to the cold. Cold stress has negative impacts on photosynthesis (Batista-Santos et al., 2011). We identified key responders that may account for the changes in photosystem-related transcription under cold stress, such as oxygen-evolving enhancer protein (OEE)-like and chlorophyll-a apoprotein P700, which were upregulated. OEE1 specifically stimulates chloroplast fructose bisphosphatase, promotes carbon fixation (Charles and Halliwell, 1981; Heide et al., 2004), and protects the light-harvesting chlorophyll-binding protein against proteolysis depending on the chlorophyll-a apoprotein P700 (Eichacker et al., 1996). Furthermore, ferredoxin was upregulated in our results. Overexpression of ferredoxin-like protein in *Oryza sativa* promotes abiotic stress tolerance by reducing the accumulation of reactive oxygen species (Huang et al., 2020). These results further support the conclusion that photosynthesis is a core *C. japonica* response to cold. In addition, most genes related to the carbon fixation pathway were upregulated, while PEPC was downregulated in Tg and Sg plants. Our previous results suggest that these genes were upregulated and played an important role in *C. japonica* at 4°C (Fan et al., 2021a), indicating that there is divergence in the regulatory mechanisms among the responses to different cold stressors. Overexpression of the PEPC gene increases the net photosynthetic rate and reduces O_2_-associated inhibition of photosynthesis under warm conditions (Zhang et al., 2014). Some studies have shown that upregulating PEPC may be important during cold acclimation (not freezing temperatures) (Sosińska and Kacperska-Palacz, 1979; Vu et al., 1995).

Circadian rhythms merit attention, including the input, central oscillator, and output pathways (Srivastava et al., 2019). The circadian system perceives temperature cues and regulates the interaction between temperature and the internal system of the plant (Srivastava et al., 2019). Most of the key members of the RESPONSE REGULATOR were downregulated in Tg and Sg *C. japonica*, e.g., PRR7, LHY, and TOC1 (Supplementary Table 7). Some transcriptome analyses have shown that the circadian clock regulates the maximum gene response to abiotic stress in Arabidopsis (Covington et al., 2008; Hubbard et al., 2009), rice (Duan et al., 2014), and barley (Habte et al., 2014). The circadian clock regulates the expression of the cold-responsive gene C-repeat binding factor 1 (CBF1) to provide resistance to cold (Maibam et al., 2013). PHYTOCHROME-INTERACTING FACTOR 3 (PIF3) acts as a negative regulator of freezing tolerance by binding to the CBF gene promoter (Jiang et al., 2017). In addition, overexpression of TOC1 (key member of the RESPONSE REGULATOR) leads to stress, as it delays stomatal closure (Legnaioli et al., 2009).

### Divergent Cold Response Pathways in *Camellia japonica*

Some genes and pathways in our transcriptome and proteome results suggest the specific response to cold stress in Tg and Sg *C. japonica*. ABA and GA are crucial hormone antagonists (Finkelstein, 2013; Shu et al., 2018). The ABA–PYR/PTL/RCAR complex is formed by ABA interacting with PYR/PYL (Rodríguez-Gacio Mdel et al., 2009). PYR/PYL was upregulated in Tg and Sg *C. japonica*. Interestingly, PP2C was downregulated in Tg, indicating the dynamics of ABA content. GID1 interacts with GA to promote the accumulation of the GA-GID1-Della protein, which stimulates plant growth (Sun et al., 2010). GID1, GID2, and DELLA were upregulated in Tg *C. japonica*, suggesting that GA synthesis increased during the cold treatment. Two DELLA proteins were downregulated in Sg *C. japonica* and displayed a complex expression trend. Our hormone profiling confirmed that GA content was upregulated in Tg *C. japonica*, but downregulated in Sg *C. japonica*. In addition, PIF3, a negative responder to cold stress (Jiang et al., 2017), was downregulated. A previous study suggested that the balance between ABA and GA regulates the development and response to stimuli in plants (Wang et al., 2006). A high level of ABA with high ratios of ABA to GA3 result in low-temperature dormancy in *C. sinensis*; however, a high IAA level with high ratios of IAA to ZR cause summer dormancy (Pan et al., 2000). The ABA/GA ratio remains low during the formation of tender shoots in *C. sinensis* during winter (Dai et al., 2021). Our hormone data revealed a similar trend, but there was divergence, as GA4 accumulated in Tg *C. japonica*, which caused the ABA/GA ratio under cold stress to be lower in Tg *C. japonica* than in Sg *C. japonica*, indicating that ABA was suppressed by accumulating GA in Tg *C. japonica* to maintain growth under cold stress. Moreover, the ABA and IAA ratio also supported this conclusion, e.g., the balance in hormones led to the divergence in cold tolerance between Tg and Sg *C. japonica*, but the underlying mechanism is unclear and worthy of further exploration.

The transcriptional and translational responses to cold stress were most evident in Tg *C. japonica*, and were strongly biased toward ribosome and protein processes. Seven ribosomal protein genes and 55 ribosomal proteins were specifically induced under cold stress in Tg *C. japonica*. Overrepresented ribosomal proteins (e.g., 40S ribosomal protein S7 and large subunit ribosomal protein L22e) are implicated in protein translation (Barkan et al., 2007) and folding (Kaur et al., 2015). In Arabidopsis, the p70 ribosomal S6 kinase homologous gene may function in the adaptation of cells to cold conditions (Mizoguchi et al., 1995). Cold temperatures affect protein translation by ubiquitinating ribosomal proteins (Gong et al., 2020). Overall, targeted changes in selected ribosomal proteins may be an integral part of the difference in freezing tolerance. In addition, plastid ribosome proteins are important components of chloroplast biogenesis. The encoding gene (TCD11) mutant of ribosomal small subunit protein S6 prevents the assembly of ribosomes in chloroplasts under low temperatures (Wang et al., 2017). Thus, these genes may regulate photosynthesis-related pathways. F-type H + -transporting ATPase was specifically induced in Tg *C. japonica*. The transgenic yeast overexpressing *ThVHAc1* exhibits increased tolerance to these abiotic stressors (Wang et al., 2020). Therefore, photosynthesis is a potential pathway regulating cold tolerance.

Although Tg *C. japonica* was more cold-tolerant, Sg *C. japonica* expressed many pathways in response to the cold. We observed transcripts and proteins related to primary and secondary metabolism that differed across the populations, with higher expression of phenylpropanoid and tryptophan biosynthetic genes and proteins in Sg *C. japonica* than in Tg *C. japonica*. Tryptophan promotes membrane association for the glycosyltransferase involved in the abiotic stress response (Ge et al., 2011). In our results, the higher expression level of glucosyltransferase (c140084.graph_c0) in Sg *C. japonica* supports this conclusion. Tryptophan protects plants from the effects of salt (Kahveci et al., 2021). In addition, a phenylpropanoid-like pathway may play a role in PsCA-enhanced chilling tolerance in cucumber (Wang B. et al., 2021), and GhANN1 improves salinity tolerance by regulating the phenylpropanoid pathway in cotton (Zhang et al., 2021).

## Conclusion

**FIGURE 7:**
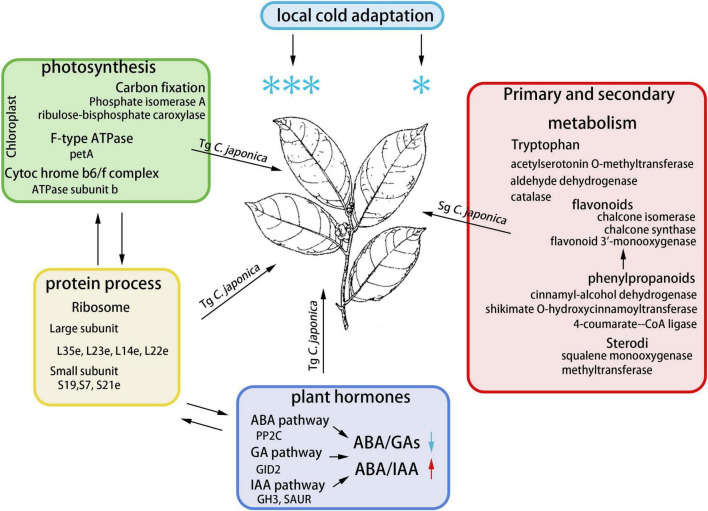
Molecular mechanism module for the response to cold in *C. japonica*. We have integrated the potential pathways and genes involved in cold resistance divergence.

Our results demonstrate divergence in the physiology, transcriptome, proteome, and plant hormones of cold responsiveness between Tg and Sg *C. japonica* (Figure 7). The results highlight the differentially expressed candidate genes involved in ribosomal protein synthesis, photosynthesis, and hormone signal transduction. The decrease in the ABA/GA ratio played an important role in improving cold tolerance in Tg *C. japonica*, which may be the main reason for the divergence of cold responsiveness. Furthermore, the ancient wild *C. japonica* (Naidong) germplasm was an exceptional source of cold-tolerant innovations with implications to improve horticultural plants.

## Materials and Methods

### Plant Material and Cold Treatments

The two populations of *C. japonica* were obtained from Qingdao (36°19′N, 120°67′E) and Zhoushan, China (30°05′N, 122°21′E) in October 2019. We selected *C. japonica* plants with more than six true leaves at similar developmental stages. The seedlings were transferred to the greenhouse at the Research Institute of Subtropical Forestry, Chinese Academy of Forestry (30°06′N, 119°96′E) for 3 months of normal culture (plastic pots: top/bottom diameter 18/9 cm, height 10 cm; temperature: 25 ± 2°C; relative humidity: 60%; soil: peat soil 80% + river sand 20%, pH: 6.0 ± 0.5; natural light). Seedlings of similar growth status from the two regions were divided into two groups. Each group was treated in a −5°C incubator for 24 h. A piece of leaf was collected every hour, physiological analyses were performed, and the sample was immediately snap-frozen in liquid N_2_, and stored at −80°C until RNA or protein extraction. Each time point contained three biological replications.

### Physiological Indices

The leaves were used to measure the oxidation index (relative electric conductivity) and osmotic index (MDA and CAT activity) to evaluate the degree of divergence in cold tolerance between Tg and Sg *C. japonica*. The CAT activity and MDA content were measured with the Malondialdehyde Content Detection kit (SolarbioBC0020, Beijing, China) and the Catalase Activity Detection kit (Solarbio^®^BC0200), following the manufacturer’s instructions. The cold-treated leaves for the relative electric conductivity assay were placed in 10 mL of deionized water and recorded as S0. The leaves were shaken at 20°C for 1 h, and S1 was determined. After boiling in water for 30 min, the samples were shaken at 20°C to cool, and S2 was determined. (S1–S0)/(S2–S0) was used to measure electrolyte leakage. The average change in the MDA contents, CAT activity, and relative electrical conductivity during each 2 h interval was calculated.

### RNA Extraction and Next-Generation Sequencing

All 24 samples (0, 4, 8, and 24 h cold treatment times of the two populations, with three biological replications each) of total RNA were extracted. Leaves were ground in liquid N_2_. Flour (100 mg) was suspended in 2.0 mL of a buffer containing RNAiso Plus (Takara^®^9108Q, Dalian, China), centrifuged at 12,000 rpm for 8 min at 4°C, and the supernatant was collected. One mL of 25:24:1 (v/v/v) phenol: chloroform: isoamyl alcohol mixture was added and centrifuged as described above to collect the supernatant. The nucleic acids were precipitated by adding 0.5 volumes of isopropyl alcohol, and ethanol was used to wash the nucleic acids. The colloidal solid was dried under ambient conditions for 15 min and dissolved in RNase-free water.

The TrueSeq RNA Sample Prep kit (Illumina Technologies, San Diego, CA, United States) was used to construct the library and to sequence the RNA of the 24 samples. mRNA was enriched using the oligo dT-bead method. After fragmentation, second-strand cDNA sample synthesis, end-repair, and ligation of the NEBNext Adaptor with a hairpin loop structure were completed. The 150–400 bp insert fragment was selected and amplified by polymerase chain reaction (PCR). The libraries were sequenced based on the Illumina HiSeq™ 2,000 platform. The raw data were stored in the NCBI database (Accession number: PRJNA769967). Adaptor sequences and low-quality reads (padj < 10) in the raw data were removed to obtain clean reads, and Trinity software (v. 2.4.0) (Grabherr et al., 2011) was used to assemble the transcriptome based on the left.fq and right.fq files. The Gene Ontology database (Ashburner et al., 2000); the Kyoto Encyclopedia of Genes and Genomes database (Kanehisa et al., 2004); the KOG (Koonin et al., 2004), the COG [(Tatusov et al., 2000); the Pfam (Finn et al., 2013) and the NR (Deng et al., 2006) databases were used to annotate the genes. Cufflinks program (Trapnell et al., 2010) was used to calculate the gene expression levels based on the FPKM values (Fragments per Kilobase of transcripts per Million mapped reads). The DEGs were identified via the DESeq (v1.10.1)]. *P*-values were corrected using the Benjamini and Hochberg approach for controlling the false discovery rate (FDR) (Love et al., 2014).

### Quantitative Proteomic Data Analysis

Proteins were extracted according to Rao et al. (2021). A BCA kit (Beyotime, Shanghai, China) was used to measure the protein concentrations, following the manufacturer’s instructions. Briefly, 0.5 M TEAB was used to dissolve the peptides, and a TMT kit (Thermo Fisher Scientific, Waltham, MA, United States) was used to label the peptides. The peptides were graded through a high pH high-performance liquid chromatography system (Agilent 300 Extend C18; Agilent Technologies Inc., Santa Clara, CA, United States). The peptides were dissolved in mobile phase A (0.1% formic acid and 2% acetonitrile) and separated on the EASY-nLC 1,000 (ThermoFisher). Mobile phase B was 0.1% formic acid and 90% acetonitrile. The liquid chromatography-electrospray ionization-tandem mass spectrometry analysis was performed according to Li J. et al. (2017). The MS/MS data were searched against our transcriptome database using MaxQuant (v1.5.2.8) (Cox and Mann, 2008). The following parameters were used for the search: Trypsin/P was the cleavage enzyme, allowing up to two missing cleavages. The mass tolerance of the precursor ions for the first search and main search was 20 and 5 ppm, respectively. The mass tolerance for the fragment ions was 0.02 Da. Carbamidomethyl on Cys was specified as a fixed modification, and methionine oxidation, protein N-terminal acetylation, and deamidation (NQ) were specified as variable modifications. The FDR was adjusted to 1% and the minimum score for the peptides was > 40. For the DEPs analysis, A *t*-test with *p* < 0.05, fold change > 1.5, or < 0.667 was considered significantly upregulated or downregulated, respectively.

The GO annotated proteome was derived from the UniProt-GOA database,^1^ and InterProScan^2^ was used to supplement the functional annotation. The proteins were classified by GO annotation based on biological processes, cellular components, and molecular functions. KAAS was used to annotate the KEGG protein database description, and the pathways were mapped in KEGG.

### Functional Enrichment and Co-expression Analyses

The functional enrichment analyses of the transcripts and proteins were performed using KOBAs (Mao et al., 2005). The R (v4.0.5) package WGCNA was used to perform the coexpression analysis using genes that contributed 25% or more of the variance (Zhang and Horvath, 2005), parameters: weighted network = unsigned, power = 8, the module genes, and Pearson’s correlation values were used to identify the correlation between the modules and the cold treatment groups. Cytoscape (v3.5.1) was used to visualize the gene networks. The expression trend analysis was performed using the R package MaSigPro (Nueda et al., 2014).

### Real-Time Polymerase Chain Reaction

The candidate genes related to the photosynthesis, ribosome, and plant hormone signaling pathways were screened. RT-qPCR of the 10 genes was carried out using the same RNA used for transcriptomic sequencing, with the same operational approaches as described before (Fan et al., 2021b). The NADPH gene was used as the reference gene (Pan et al., 2020). The relative expression level was determined by the 2^–ΔΔCt^ method (Livak and Schmittgen, 2001). The expression level of Tg-CK was set to 1, and that of other samples was determined relative to Tg-CK. The primer sequences are shown in Supplementary Table 14.

### Quantification of Phytohormones

Leaves were ground in liquid nitrogen, and 10 ml of methanol (80%, v/v) was added to extract the hormones. The mixture was centrifuged at 3,600 rpm for 11 min at 4°C, the supernatant was collected, and passed through a Chromosep C18 column (C18 Sep-Park Cartridge, Waters Corp., Milford, MA, United States) and dried. An enzyme-linked immunosorbent assay (ELISA) was used to quantify the hormones according to Chen et al. (2021). Briefly, the mouse antigens and antibodies and the horseradish peroxidase substrate were produced at China Agricultural University (Beijing, China). Coating buffer (1.5 g/L Na_2_CO_3_, 2.93 g/L NaHCO_3_, and 0.02 g/L NaN_3_) and the antigens were incubated at 37°C for 4 h for ZR, GAs, BR, and ABA and 24 h for the IAA assay at 4°C. The plates were washed with PBS. An ELISA reader (model EL310, Bio-TEK, Winooski, VT, United States) was used to check color. Each group contained three biological replicates and three technical repetitions.

### Statistical Analysis

The statistical analysis was performed via SPSS (v21.0) software (IBM Corp., Chicago, IL, United States). The Student’s *t*-test was used to calculate differences in the mean values. A *p*-value < 0.05 was considered significant. The data are presented as mean ± standard deviation of three independent experiments.

## Data Availability Statement

The original contributions presented in the study are publicly available. This data can be found here: National Center for Biotechnology Information (NCBI) BioProject database under accession number PRJNA769967.

## Author Contributions

MF conducted the data analysis and wrote the manuscript. XL and HY conceived and designed the experiments. YZ, MY, and SW performed the experiments. WL and JL contributed plant material. All authors have read and agreed to the published version of the manuscript.

## Conflict of Interest

The authors declare that the research was conducted in the absence of any commercial or financial relationships that could be construed as a potential conflict of interest.

## Publisher’s Note

All claims expressed in this article are solely those of the authors and do not necessarily represent those of their affiliated organizations, or those of the publisher, the editors and the reviewers. Any product that may be evaluated in this article, or claim that may be made by its manufacturer, is not guaranteed or endorsed by the publisher.

## Footnotes

1
http://www.ebi.ac.uk/GOA/

2
www.ebi.ac.uk/interpro/

## Supplementary Material

Supplementary Figure 1 Phenotypic changes in leaves under the cold treatment. The dark area represents the accumulation of cold damage.

Supplementary Figure 2 Differentially expressed transcript divergence between Tg and Sg *C. japonica* under cold stress. Venn diagram represents the number of differentially expressed genes, and CK, 4 h, 8 h, 24 h indicate Tg-CK vs. Sg-CK, Tg-4 h vs. Sg-4 h, Tg-8 h vs. Sg-8 h, and Tg-24 h vs. Sg-24 h, respectively.

Supplementary Figure 3 Expression of candidate genes in response to cold stress as determined by RT-qPCR **(A)** and the transcriptome **(B)**.

Supplementary Table 1 The raw data of physiology index.

Supplementary Table 2 The sequence data of transcriptomics and proteomics.

Supplementary Table 3 The enrichment KEGG pathways of Tg vs. Sg.

Supplementary Table 4 The enrichment GO terms of Tg vs. Sg.

Supplementary Table 5 The functional enrichment pathways of Tg and Sg under cold stress.

Supplementary Table 6 The functional enrichment pathways of Tg vs. Sg under cold stress.

Supplementary Table 7 The enrichment GO terms of WGCNA.

Supplementary Table 8 The enrichment KEGG pathways of WGCNA.

Supplementary Table 9 The annotation of cold genes related to photosynthesis.

Supplementary Table 10 The annotation of Carbon fixation.

Supplementary Table 11 The annotation of ribosome.

Supplementary Table 12 The raw data of plant hormone measurement.

Supplementary Table 13 The temperature of Qingdao and Zhoushan.

Supplementary Table 14 The primer and RAW data of RT-qPCR.
