# Supplementary figures and images for: Multi-Approach Analysis Reveals Pathways of Cold Tolerance Divergence in Camellia japonica
Source: Front Plant Sci. 2022 Feb 25;13:811791. doi: 10.3389/fpls.2022.811791 (PMC8914472; doi:10.3389/fpls.2022.811791)

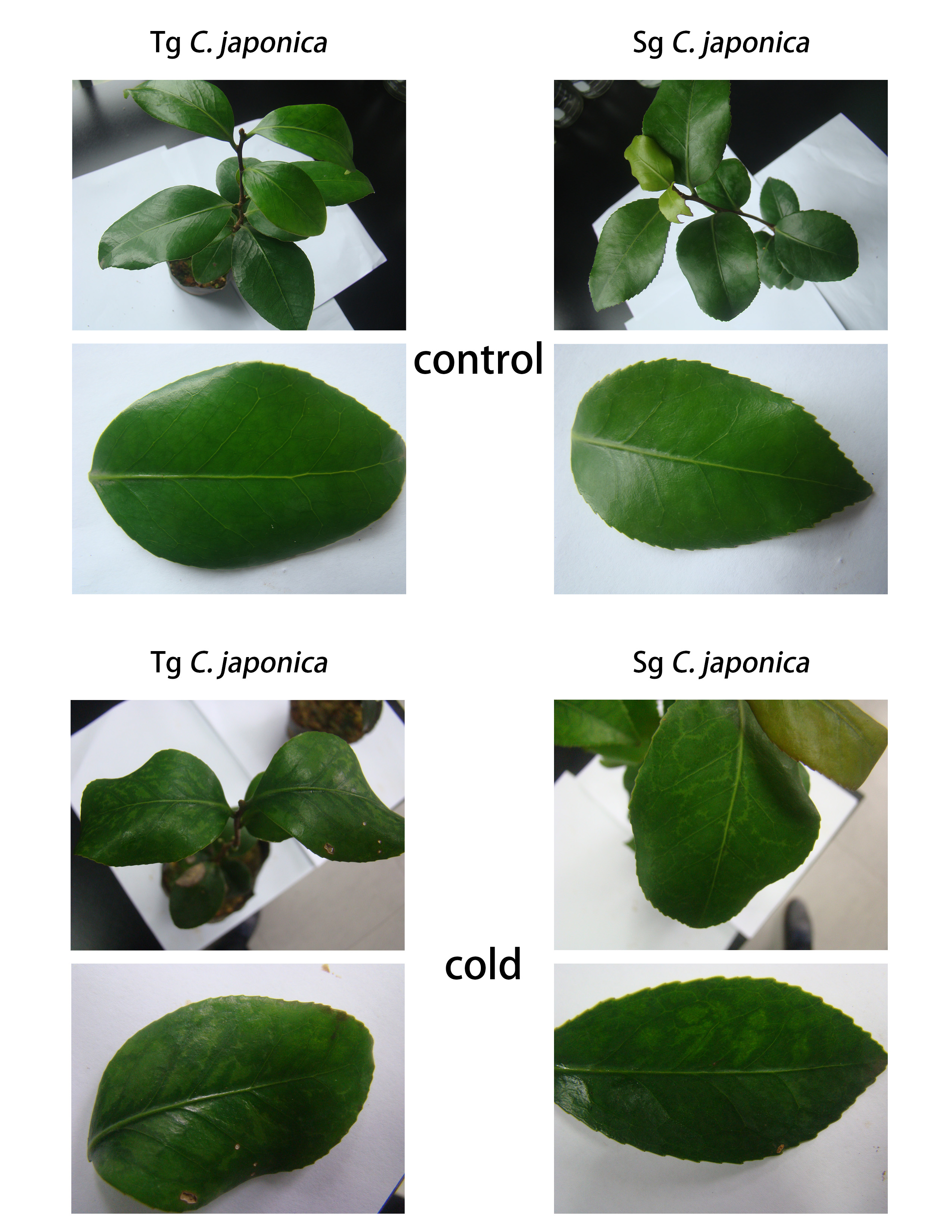

Supplement: Supplementary Figure 1 — Phenotypic changes in leaves under the cold treatment. The dark area represents the accumulation of cold damage. [file Image_1.JPEG]

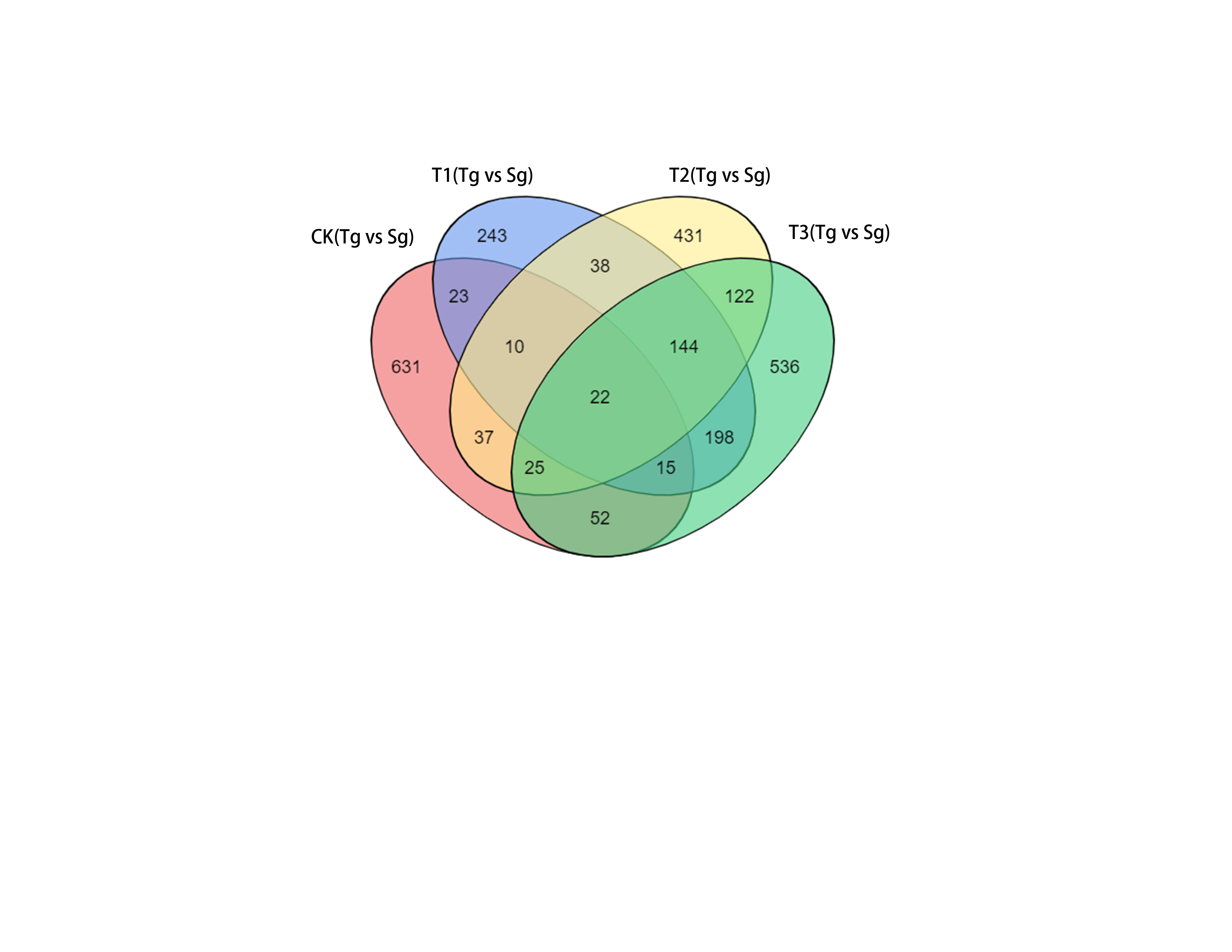

Supplement: Supplementary Figure 2 — Differentially expressed transcript divergence between Tg and Sg C. japonica under cold stress. Venn diagram represents the number of differentially expressed genes, and CK, 4 h, 8 h, 24 h indicate Tg-CK vs. Sg-CK, Tg-4 h vs. Sg-4 h, Tg-8 h vs. Sg-8 h, and Tg-24 h vs. Sg-24 h, respectively. [file Image_2.JPEG]

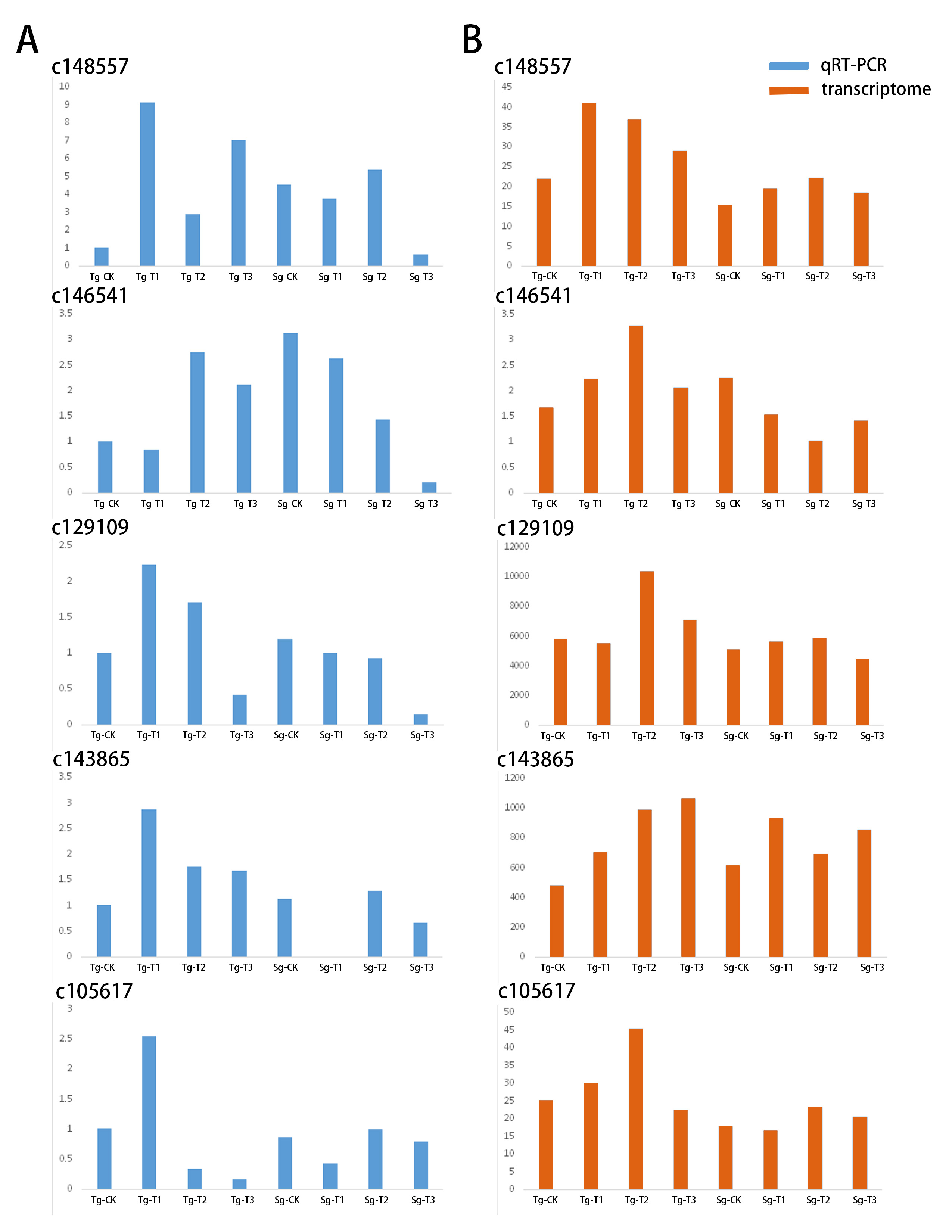

Supplement: Supplementary Figure 3 — Expression of candidate genes in response to cold stress as determined by RT-qPCR (A) and the transcriptome (B). [file Image_3.JPEG]
